# Risk Factors and Modifiers for Cardiovascular Disease Assessment of Patients with Heterozygous Familial Hypercholesterolaemia

**DOI:** 10.3390/jcm13082270

**Published:** 2024-04-14

**Authors:** Richard Malone, Sarah Savage, Vivion Crowley, Martina Hennessy, Patricia O’Connor, Cormac Kennedy

**Affiliations:** 1 School of Medicine, Trinity College Dublin, D08 W9RT Dublin, Ireland; maloneri@tcd.ie (R.M.); mhenness@tcd.ie (M.H.); poconnor@stjames.ie (P.O.); 2 Department of Biochemistry Department, St James’s Hospital, D08 W9RT Dublin, Ireland; ssavage@stjames.ie (S.S.); vcrowley@stjames.ie (V.C.); 3 Department of Pharmacology & Therapeutics, St James’s Hospital, D08 W9RT Dublin, Ireland

**Keywords:** familial hypercholesterolemia, cardiovascular risk, assessment, risk modifier, risk factor

## Abstract

**Background**: The assessment of the risk of cardiovascular disease (CVD) in patients with heterozygous familial hypercholesterolemia (HeFH) is determined by conventional risk factors. However, factors modifying CVD, or risk modifiers, beyond conventional risk factors may inform their CVD risk assessment and the subsequent use of new therapies. This work identifies and characterises patients within a lipid clinic cohort with regards to conventional CVD risk factors and risk modifiers with a focus on those with HeFH. **Methods:** A study of consecutive adult patients attending our specialist lipid clinic was performed over a six-month period. The patient data recorded included demographics, clinical characteristics, risk factors and risk modifiers, biochemical profiles and genetic testing results. Risk modifiers were identified based on ESC/EAS guidance, and those with HeFH were compared to those without. **Results:** A total of 370 patients were included. Of these, 98 HeFH patients were identified (26%). Then, 52% of HeFH patients were stratified into the very-high risk category due to the presence of CVD risk factors. Risk modifiers were present in 73%. These included a family history of premature CVD (56%), obesity (28%), a sedentary lifestyle (13%) and a major psychiatric disorder (12%). Compared to the rest of the cohort, those with HeFH were less likely to have hypertension and more likely to have a family history of premature CVD. **Conclusions:** Half of patients with HeFH are categorised as having very high CV risk. Consideration of risk modifiers, particularly a family history of premature CV disease, increases this very-high-risk category further. This may have implications for the clinical application and access to novel treatments.

## 1. Introduction

Heterozygous Familial Hypercholesterolemia (HeFH) is a monogenetic lipid disorder caused by mutation in one or more genes governing the clearance of low-density lipoprotein cholesterol (LDL-C). This is a common genetic disorder (1 in 200–250) affecting 14–34 million people worldwide, yet only a minority of cases are recognised and treated accordingly [1]. The impairment in LDL-C removal gives rise to lifelong elevations in LDL-C levels, accelerating atherosclerosis and conferring a minimum grading of high cardiovascular disease (CVD) risk as per the European Society of Cardiology (ESC) and European Atherosclerosis Society (EAS) guidelines [1]. Clinical diagnosis of FH is facilitated by the use of validated diagnostic criteria such as the Dutch Lipid Clinic Network (DLCN). A clinical diagnosis of the FH phenotype can be confirmed genetically thereafter to identify the causative mutation [2]. However, it has been noted that up to 60% of patients that demonstrate a FH phenotype fail to demonstrate a detectable genetic mutation [2,3]. Similarly, the accurate estimation of CVD risk in HeFH remains challenging.

Global CVD risk estimation is a function of validated tools such as the Framingham Risk Score or the European SCORE which are derived from general populations and integrate multiple factors such as age, sex, smoking status, blood pressure and lipid profile [4]. Established risk calculators derived from non-HF populations are deemed inappropriate in European, North American and Asian guidelines in predicting CVD risk among FH populations [1,5,6]. Within the European context, other major risk factors highlighted in ESC guidance include severe chronic kidney disease (CKD), diabetes and, more recently, a raised lipoprotein(a) level [1]. CVD risk can be further refined through a consideration of factors modifying CVD, or risk modifiers, and adjunctive risk assessment techniques such as computed tomography coronary artery calcification scoring [1,7]. This concept of risk modifiers has been developed further in the more recent ESC guidelines on CVD prevention and encompass lifestyle factors, socioeconomic determinants and comorbid conditions [7].

Appropriate lipid lowering therapy selection, guided by an LDL-C goal, is currently a function of accurate CVD risk assessment. The utility of established CVD risk calculators within HeFH populations, as discussed, is limited, due to a trend towards risk underestimation [8]. Furthermore, significant heterogeneity in CVD incidence within FH populations has been observed. This heterogeneity exists despite comparable LDL-C levels and mutation variants [9,10]. Proportions of HeFH cohorts with significantly elevated LDL-C levels do not manifest symptomatic CVD while others develop premature CVD [11].

As per current ESC guidance, HeFH patients with the presence of established atherosclerotic cardiovascular disease (ASCVD) or one other ‘major risk factor’ encompasses the criteria for further stratification into the very-high-risk category [1]. The ramifications of distinguishing between high and very-high CVD risk among HeFH patients is evident when considering that current ESC guidance suggests the cost-effectiveness of PCSK9 inhibitors is limited to very-high-risk HeFH patients that have failed to achieve lipid treatment goals with a maximally tolerated dose of statins in addition to ezetimibe. It is hypothesised that a multifaceted dynamic between genetic and environmental risk modifiers, which extends beyond standard risk factors, will account for the heterogeneity in CVD outcomes observed within HeFH populations [12]. Given the challenges and limitations in current HeFH risk assessment approaches, the utility of incorporating ESC risk modifiers to refine CV risk warrants investigation.

The aims of this study included:

1. Determine the clinical characteristics of a cohort of patients attending a Lipid Clinic over a six-month period.
2. Identify HeFH patients with either a clinical diagnosis or genetically confirmed diagnosis.
3. Profile the prevalence and both conventional CVD risk factors and risk modifiers as per current ESC guidance in the cohort with comparative HeFH subgroup analysis.

## 2. Patients and Methods

### 2.1. Setting and Study Design

St James’s Hospital (SJH) is a tertiary academic hospital in Dublin, Ireland. The Lipid Clinic receives patient referrals warranting specialist input for familial hypercholesterolemia screening, abnormal lipid profiles, statin intolerance, high CVD risk and treatment optimisation, including PCSK9i access. This study was initiated in July 2022 and encompassed the retrospective chart review of consecutive adult patients attending the Lipid Clinic over a six-month period (July 2021–December 2021).

### 2.2. Consent Process and Anonymisation

The dataset was subject to anonymization, encryption and password protection. Compliance with the European Union General Data Protection Regulations was adheredthroughout the study period and reviewed by the hospital’s data protection office. Tallaght University Hospital Research Ethics Committee granted ethical approval for this study.

### 2.3. Data Collection and Extraction

The primary data points of interest are noted in Table 1. Patient data were reviewed retrospectively from the Electronic Patient Record (EPR). Patient charts were also subject to review where the EPR was insufficient to complete data fields. Diagnostic tests performed outside of the study window were utilised where applicable such as for pre-existing genetic testing results. Similarly, patients’ baseline lipid profiles were obtained from general practitioner referral letters or from clinic bloods for first time patients attending the clinic where no treatment had been initiated. The validated FH Wales ‘LDL-C Estimator’ tool was used to calculate LDL-C levels in instances where pre-treatment or on treatment LDL-C levels were not available [13]. Further information concerning laboratory test ranges and procedures is available at the SJH website. Data obtained and recorded in patients notes as part of the standardised lipid clinic attendance included:

- Patient’s height, weight and body mass index (BMI) as assessed by nursing staff at each clinic visit and recorded into patient charts. Exceptions included, but were not limited to, telephone consultations and/or the patient’s refusal or inability to ambulate (wheelchair users).
- Family history of CVD and age of CVD to ascertain whether premature CVD was present.
- Physical examination findings including weight, height, BMI, and lipid deposition (xanthoma, xanthelasma and corneal arcus) which was assessed by senior clinicians.
- Patient physical activity was noted as part of standard history taking in the clinic. Categories included the following: walking, running, cycling, gym, cardio, resistance training, mixed, team-based sports, none and other.

### 2.4. Definitions

A diagnosis of HeFH was assigned to those patients that met the criteria for a clinical and/or genetically confirmed diagnosis. A clinical diagnosis of the FH-phenotype (FH-P) was conducted in accordance with the DLCN Score as detailed in the data analysis section. A genetically confirmed diagnosis was attributed to patients for whom a positive mutation was detected in the low-density lipoprotein receptor (LDL-R), apolipoprotein B (ApoB) or proprotein convertase subtilisin/kexin 9 (PCSK9) genes. Genetic testing methods included Next-Generation sequencing (NGS) and/or multiplex ligation dependent probe amplification (MLPA). Patients that met the both the clinical and genetic criteria were classified as having the FH phenotype and genotype (FH-PG). As per ESC guidance, the factors modifying systemic coronary risk estimation risks include social deprivation, obesity, physical inactivity, psychosocial stress, family history of premature CVD, chronic immune mediated inflammatory disorder, major psychiatric disorders, HIV treatment, atrial fibrillation, left ventricular hypertrophy, chronic kidney disease, obstructive sleep apnoea and non-alcoholic fatty liver disease [1]. The presence of CVD was denoted by medical records listing any of the following diagnoses: myocardial infarction, percutaneous coronary intervention, coronary artery bypass graft, unstable angina, stable angina, ischaemic stroke, transient ischaemic attack, carotid stenosis, arterial revascularisation, peripheral vascular disease, coronary artery disease, valvular disease, atrial fibrillation, other arrhythmias or left ventricular hypertrophy. Major psychiatric diagnoses included depression, schizophrenia, generalized anxiety disorder and bipolar affective disorder as recorded in the patient’s clinical notes. Chronic kidney disease stages 3, 4 and 5 were attributed to patients that demonstrated, an estimated glomerular filtration rate (calculated using the Modification of Diet in Renal Disease formula) in the ranges of 30–59 mL/min, 15–29 mL/min, 0–14 mL/min, respectively. Such measurements were evident for at least a three-month period. Patients were classified as having an inflammatory disease where their records stated a given chronic immune mediated inflammatory diagnosis such as rheumatoid arthritis or inflammatory bowel disease. Premature CVD was defined as CVD disease occurring before the age of 55 years or 60 years in males and females, respectively. A family history of premature CVD was classified as premature CVD occurring in a first-degree relative. A sedentary lifestyle was attributed to patients that self-reported as not engaging in any of the following forms of exercise; walking, running, cycling, gym, cardio, resistance training, mixed training, team-based sports or ‘other’.

### 2.5. Data Analysis

The dataset was coded and organized to enable analysis. Patients were subject to the DLCN scoring system. This evidence-based points system assigns points for genetic investigation results, LDL-C levels, lipid deposition findings on physical exam, family history and the presence of premature cardiovascular disease. The cumulative score stratifies patients into levels of probability of having FH (definite, probable, possible or unlikely). This score was quoted from the patient’s notes, where listed, or otherwise calculated from patient’s data using an online calculator tool [14]. Patients were classified as obese where their listed or calculated BMI was ≥30.

### 2.6. Statistical Analysis

Normally distributed continuous data are presented as mean with standard deviation. Categorical data are presented as frequencies with percentages. The two HeFH subgroups (FH-P and FH-PG) were subject to comparative analysis with the general study cohort. Statistical analysis was performed with Windows Excel Version 2401 (Microsoft Corporation, Washington, DC, USA). Statistical methods for performing group comparative analysis were via standard two-tailed *t* test (continuous variables) and Chi squared test (categorical variables). As standard, the minimum level of statistical significance was 5% (*p* < 0.05).

## 3. Results

### 3.1. Cohort Characteristics

A total of 370 patients were included in the study. Table 2 details the demographics of the study cohort and HeFH subgroups. In total, 26% of patients were attending the clinic for the first time (*n* = 98). The cohort had a mean age of 49 ± 14 years and was predominantly female (55%). There was no significant difference in gender across the study groups.

Table 3 details the prevalence of CVD risk factors and risk modifiers to the study cohort and HeFH subgroups. CVD risk factors included hypertension (34%), established CVD (23%), tobacco smoking (15%) and type 2 diabetes mellitus (11%).

The most prevalent CV risk modifiers (Table 1) were noted as a family history of premature CVD (39%), obesity (33%), a sedentary lifestyle (17%), a major psychiatric disorder (15%), non-alcoholic fatty liver disease (12%) and inflammatory disease (9%). Chronic kidney disease stages 3–5, obstructive sleep apnoea and psychosocial stress shared an equal prevalence of 2%. Atrial fibrillation, left ventricular hypertrophy and HIV were present in 1%. As a tertiary referral centre for haematological conditions, post-haematopoietic stem cell transplant patients were 2% of the cohort.

### 3.2. Heterozygous Familial Hypercholesterolemia Phenotype (FH-P) and Phenotype and Genotype (FH-PG)

**Figure 1:**
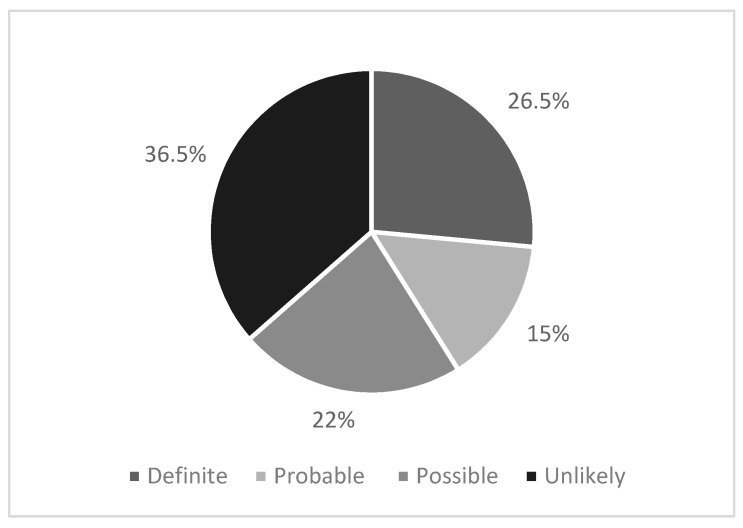
Dutch Lipid Clinic Network Score for the cohort.

Upon review, 26% of the cohort (*n* = 98) met the criteria for a phenotypic diagnosis of HeFH as per the DLCN. Figure 1 illustrates the DLCN scoring profile of the cohort. These patients were predominantly female (61%). Compared with the rest of the cohort, this group were younger, with an average age of 46 ± 13 years (*p* < 0.001).

Furthermore, 58% of the FH-P patients met the criteria for a dual phenotype/genotype diagnosis (FH-PG) following the identification of an associated mutation.

**Figure 2:**
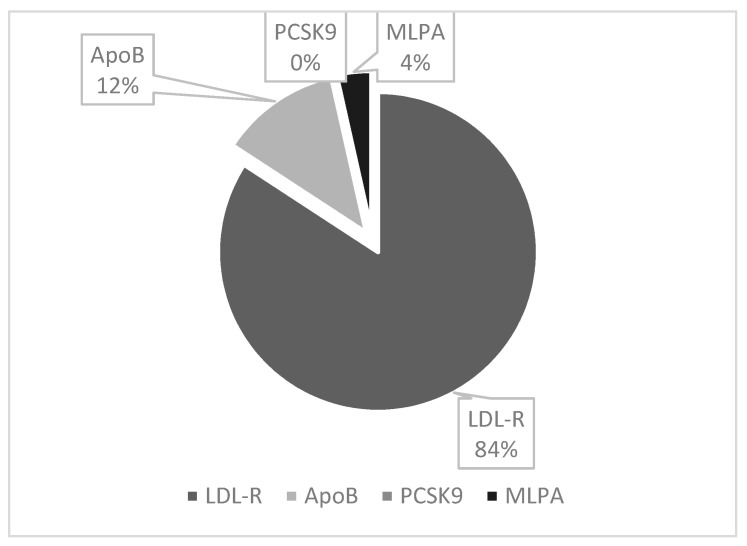
Patients with a mutation detected (*n =* 57).

FH-PG patients were predominantly female (61%) and younger than the clinic cohort at 41 ± 13 years (*p* < 0.001). Figure 2 illustrates the gene mutations detected.

### 3.3. HeFH Subgroup Risk Assessment

**Figure 3:**
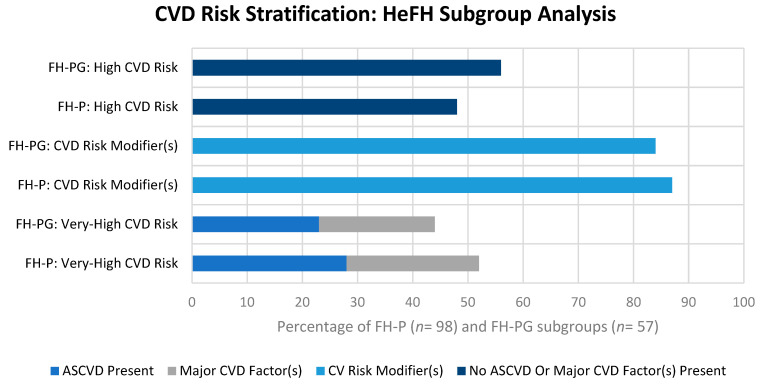
CVD Risk Stratification: HeFH Subgroup Analysis.

**Figure 4:**
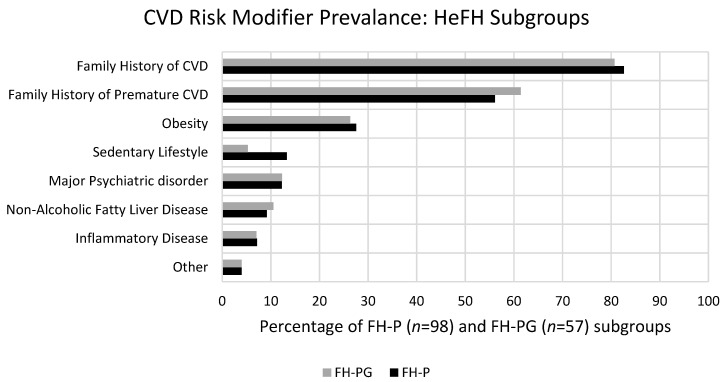
CVD Risk Modifier Prevalence: HeFH Subgroups.

Figure 3 illustrates the stratification of the HeFH subgroups into the high and very-high CVD risk categories with a comparison of respective CVD risk modifier profiles. Figure 4 further illustrates the comparative prevalence of individual CVD risk modifiers among the HeFH subgroups.

Moreover, 52% of patients within the FH-P subgroup were stratified into the very-high-risk category due to the presence of established ASCVD (28%) or the presence of at least one major CVD factor (24%). CVD risk modifiers were present in 87% of the FH-P group. The most common of these modifiers included a family history of CVD (83%), a family history of premature CVD (56%), obesity (28%), physical inactivity (13%), major psychiatric disorders (12%), NAFLD (9%) and inflammatory conditions (7%).

In addition, 44% of patients within the FH-PG category were stratified into the very-high-risk category due to the presence of established ASCVD (23%) or due to the presence of at least one major CVD Factor (21%). CVD risk modifiers were present in 84% of the FH-P group. The most common of these modifiers included a family history of CVD (81%), a family history of premature CVD (61%), obesity (26%), major psychiatric disorders (12%), NAFLD (11%), inflammatory conditions (7%) and physical inactivity (5%). In the absence of major CV risk factors, 33% of HeFH patients would be stratified into the very-high-risk category due to the presence of one or more risk modifiers. Overall, 85% of HeFH patients were stratified into the very-high CVD risk category.

### 3.4. Physical Examination Findings

Overall, 162 patients in our cohort (44%) demonstrated one or more clinical signs of lipid deposition. This was xanthoma in 17%, xanthelasma in 5% and corneal arcus in 22%. Both FH subgroups demonstrated comparatively higher rates of one or more clinical signs. This was the case for 67% in the FH-P group with xanthoma present in 48% (*p* < 0.001) for the comparison with the rest of the cohort), xanthelasma in 8% (*p* = 0.11), and corneal arcus in 42% (*p* < 0.001). Moreover, 54% of patients within the FH-PG group had these findings. This was 35% for xanthoma (*p* < 0.001), 4% for xanthelasma (*p* = 0.55) and 37% corneal arcus (*p* < 0.001).

## 4. Discussion

This pragmatic study identified a HeFH population within a cohort of patients attending a specialist lipid clinic with comparative analysis of their clinical characteristics, CVD risk factors and risk modifier profile. In the clinic, typical HeFH patients were characterised by a younger age and a significantly elevated baseline lipid profile relative to non-HeFH patients. The most common comorbidities, affecting approximately 1 in 4 patients, included established CVD, obesity and hypertension. Less frequent but notable comorbidities, affecting approximately 1 in 10 patients, included major psychiatric disorder, NAFLD, type 2 diabetes mellitus and an inflammatory disease. Excluding hypertension, the frequencies of these comorbidities were comparable to non-HeFH patients. Over half of the HeFH patients had a positive family history of premature CVD. As expected, the significant majority of HeFH exhibit the clinical signs of lipid deposition during physical examination, with two-thirds of patients assigned a definite FH or probable FH by their DLCN score.

In addition, 26% of the cohort met the criteria for a clinical diagnosis of HeFH whereas 15% met the criteria for a dual phenotype and genotype diagnosis. This finding also highlights the discrepancy in the translation of phenotypic diagnoses into positive genetic testing results. This is consistent with prior research that reports such a figure may be as high as 60% [3]. This may reflect unidentified pathogenic mutations in both known and unknown FH genes or a polygenic process yet to be elucidated [15]. In either case, this highlights the current clinical importance and utility of the validated diagnostic tools such as the DLCN Score.

The accurate stratification of HeFH patients into the very-high-risk tier following diagnosis is a fundamental function of the lipid clinic, particularly in ensuring the judicious access of more potent and costly therapeutic options, for example, the PCSK9 inhibitors. The presence of established CVD or one other ‘major risk factor’ encompasses the criteria for further stratification into the very-high-risk category as previously discussed. Half of the HeFH patients were classified as very-high for CVD risk following risk factor analysis, increasing to 85% if risk modifiers were included. The factors and modifiers of notable prevalence warrant further discussion.

The prevalence of a family history of premature CVD was significantly elevated in both FH-P (56%) and FH-PG (61%) subgroups relative to the general cohort rate of 39%. This is in keeping with existing research. A previous cohort study of HeFH patients (*n* = 821) undergoing lipid-lowering treatment demonstrated a positive association between patients’ progression to premature CVD in those with a family history of the same [16]. A family-based HeFH study found a significantly elevated standardised mortality ratio in HeFH patients with a family history of premature CAD compared to those without such a family history [17]. This proved to be the only risk modifier that demonstrated statistical significance in both HeFH subgroups relative to the cohort.

Obesity encompassed a significant comorbidity in our study, affecting 1 in 4 HeFH patients. These findings reflect the ongoing challenges posed by the obesity epidemic. Irish obesity rates are currently reported at approximately 22% for females and 25% for males [18]. Standard consultations in the lipid clinic encompass BMI assessment and a review of patient diets, including the profiling of the typical daily diet in addition to screening for the nature and frequency of suboptimal food choices. Verbal counselling and guidance leaflets are issued accordingly. The clinic has access to expert input from clinic nutritionists on referral. These findings reinforce the clinical need for robust public health intervention. Ideally, the development of services and associated infrastructure to provide nutrition, exercise and weight loss programs may prove efficient in tackling these issues [18].

Hypertension has continually proven to be an independent modifier of CVD risk in HeFH patients [10]. Our HeFH subgroups demonstrated significantly lower comparative rates of hypertension (FH-P: 26%, FH-PG; 19%). This may reflect the statistically significant younger age profile of the HeFH subgroups as detailed in Table 2.

The rates of sedentary lifestyle in the FH-P and FH-PG subgroups were 13% and 5%, respectively. This rate was lower compared to the general cohort (17%) but only proved significant in the genetically confirmed HeFH patients. Perhaps this reflects the impact of a confirmed genetic diagnosis or the awareness of the cardioprotective effect of exercise in families with CVD. In the clinic, patients self-report on the nature and frequency of physical activity as standard. Patients deemed to be sedentary are counselled in line with the ESC guidance on exercise [1].

In total, 12% of HeFH patients reported a major psychiatric disorder. Existing research has demonstrated that conditions, such as depression, bipolar disorder and schizophrenia, are associated with premature mortality compared with the general population [19,20]. ASVCD is a determinant in this regard, with meta-analysis demonstrating a 78% increase in ASCVD risk and an 85% increase in resulting mortality [21]. Research concerning major psychiatric disorders in HeFH has already been noted to be severely limited [22].

Briefly, in terms of other risk modifiers identified in this cohort, NAFLD and inflammatory conditions are of interest. There is a paucity of evidence regarding the interaction of NAFLD and HeFH, and so the information here adds to a previous small study [23]. Similarly, while the interaction of chronic immune-mediated inflammatory diseases such as systemic lupus erythematosus, rheumatoid arthritis and inflammatory bowel diseases, with ASCVD has been reported, their significance in HeFH cohorts is unknown [24,25,26]. Inflammation in FH patients has been implicated as a synergistic element in atherosclerosis progression [27]. However, the use of newer therapies means these conditions are often adequately managed, and the increase in CVD risk attributable to them may be modest.

### HeFH CV Risk Assessment: Current Limitations and Future Opportunities

This study has demonstrated the prevalence of a risk modifier in a cohort of HeFH patients. How this information may be used in CVD risk stratification is complex. The prognostic utility of conventional CVD factors and risk modifiers as applied to HeFH populations remains problematic. Observational studies of FH populations have been conducted, albeit with varying methodological quality, aiming to estimate CVD risk [28,29]. These have failed to demonstrate a consensus of the prognostic weighting of FH on CVD risk. Studies focusing on FH patient with pre-existing CVD or those which include homozygous FH patients has given rise to overestimation bias [12]. Conversely, risk underestimation has arisen in studies where fatal CVD events are chosen as the primary outcome measure. Research methods involving the pooling of treated with untreated HeFH case control series are problematic. Such control patients may harbour unknown HeFH given the high false negative rate on genetic testing [12]. Data derived from HeFH registries primarily concerns patients, treated or otherwise, that have no CV events [12].

Bianconi et al., acknowledge that CVD risk varies significantly among HeFH patients. They have highlighted that the additional CVD risk modifiers are encompassed within three primary domains: genetic parameters other than HeFH causing mutations, indices of vascular injury and biomarkers [12]. Genetic parameters beyond the FH-causing mutations under consideration include telomere length in somatic cells, single nucleotide polymorphisms and other genetic variants. Biomarkers of interest reflecting levels of inflammation, oxidation and aberrant haemostasis have been identified within FH patients via proteomic techniques. Structural and functional assessments of vascular injury include ultrasound carotid intima-media thickness and computer tomography based coronary artery calcium (CAC) quantification.

Ultimately, the quantification of the dynamic interplay between these domains may yield a more refined FH risk assessment and, therefore, personalised treatment optimisation. Given the multiple inputs and complexity of this assessment, artificial intelligence may have a role to play in appropriately stratifying risk in HeFH patients [30]. 

## 5. Strengths and Limitations

The strengths of this study included a pragmatic design that yielded an authentic demonstration of contemporary lipid clinic patients in addition to the assessment of their CVD risk. The clinic has access to FH genetic testing, and, therefore, all patients requiring genetic analysis receive it; a limitation in other jurisdictions. The primary limitation of this study included its observational nature. Certain risk modifiers of interest such as social deprivation are not routinely screened during lipid clinic consultations.

## 6. Conclusions

This study identified a HeFH population within a cohort of patients attending a specialist lipid clinic. Half of patients with HeFH are categorised as having very high CV risk. Consideration of risk modifiers, particularly a family history of premature CV disease, may increase those in the very-high-risk category further. This has implications for the application of, and access to, novel treatments.

## Figures and Tables

**Table 1:** Data points.

Demographics	Gender	Age	Height	Weight
	BMI	Referral Details		
Comorbidities	Hypertension	Established CVD	T2DM	Obesity
	NAFLD	Inflammatory Disease	HIV	Post HSCT
	CKD 3–5	OSA	Major Psychiatric Disorder	
Lifestyle Factors	Smoking Status	Psychosocial Stress	Physical Activity	
Lipid Lowering Therapy	Generic Name	Dose	Frequency	
Laboratory Investigations	Lipid Profiles	LFTs	TSH	FH Genetic Testing
	HbA1c	CK	Renal Function	
Imaging	Coronary Artery Calcium Score	Carotid Plaque on Ultrasound		
Physical Examination Findings	Xanthoma	Xanthelasma	Corneal Arcus	

BMI: body mass index, CK: creatine kinase, CKD: 3–5 chronic kidney disease stages 3–5, CVD: cardiovascular disease, FH: familial hypercholesterolemia, HIV: human immunodeficiency virus, HSCT: haematopoietic stem cell transplant, LFTs: liver functions tests, NAFLD: non-alcoholic fatty liver disease, OSA: obstructive sleep apnoea, T2DM: type 2 diabetes mellitus, TSH: thyroid stimulating hormone.

**Table 2:** Patient Characteristics.

	Study Cohort	FH-P	*p*	FH-P/G	*p*
Number of patients	370	98 (26%)	-	57 (15%)	-
Age	49.43 ± 13.59	45.70 ± 13.13	0.001	40.93 ± 13.15	0.000
Male	168 (45%)	38 (39%)	0.124	22 (39%)	0.262
Female	202 (55%)	60 (61%)		35 (61%)	

FH-P: Familial Hypercholesterolemia phenotype, FH-PG: Familial Hypercholesterolemia phenotype and genotype.

**Table 3:** CVD Risk Factors and Modifiers.

	Whole Cohort *n =* 370	FH-P *n =* 98	*p*	FH-PG *n =* 57	*p*
Smoking Status					
Never Smoker	208 (56%)	53 (54%)	0.619	36 (63%)	0.251
Current Smoker	57 (15%)	17 (17%)	0.535	8 (14%)	0.755
Ex-smoker	100 (27%)	27 (28%)	0.892	12 (21%)	0.269
Lipid Profile					
Baseline TC	7.95 ± 1.92	8.99 ± 2.02	0.000	9.49 ± 2.39	0.000
Baseline LDL-C	5.57 ± 1.83	6.69 ± 1.88	0.000	7.22 ± 2.15	0.000
Baseline TG	3.68 ± 4.96	2.02 ± 2.33	0.000	2.02 ± 3.02	0.001
Lp (a) > 70 nmol/L	127 (34%)	33 (34%)	0.874	16 (28%)	0.280
Lp (a) > 430 nmol/L	18 (5%)	3 (3%)	0.333	2 (4%)	0.605
Co-morbidities					
Established CVD	85 (23%)	27 (28%)	0.209	13 (23%)	0.974
Hypertension	125 (34%)	25 (26%)	0.043	11 (19%)	0.012
Type 2 Diabetes Mellitus	42 (11%)	7 (7%)	0.126	4 (7%)	0.262
Family History of Premature CVD	145 (39%)	55 (56%)	0.000	35 (61%)	0.000
Co-morbidities					
Obesity	121 (33%)	27 (28%)	0.205	15 (26%)	0.264
Non-Alcoholic Fatty Liver Disease	43 (12%)	9 (9%)	0.380	6 (11%)	0.779
Atrial Fibrillation	4 (1%)	1 (1%)	0.946	-	0.391
Left Ventricular Hypertrophy	3 (1%)	-	0.297	-	0.458
Inflammatory Disease	35 (9%)	7 (7%)	0.361	4 (7%)	0.493
HIV	3 (1%)	-	0.297	-	0.458
Post Haematopoietic Stem Cell Transplant	8 (2%)	-	0.086	-	0.222
Chronic Kidney Disease Stage 3–5	9 (2%)	-	0.068	-	0.195
Obstructive Sleep Apnoea	7 (2%)	2 (2%)	0.900	2 (4%)	0.330
Major Psychiatric Disorder	55 (15%)	12 (12%)	0.395	7 (12%)	0.551
Lifestyle Factors					
Psychosocial Stress	7 (2%)	2 (2%)	0.900	-	0.254
Sedentary Lifestyle	62 (17%)	13 (13%)	0.280	3 (5%)	0.012

CVD: Cardiovascular Disease, FH-P: Familial Hypercholesterolemia Phenotype, FH-PG: Familial Hypercholesterolemia Phenotype and Genotype, HIV: Human Immunodeficiency Virus, LDL-C: Low-Density Lipoprotein C (mmol/L), Lp(a) lipoprotein (a), TC: Total Cholesterol (mmol/L), TG: Triglycerides (mmol/L).

## Data Availability

The anonymised data supporting the conclusions of this article are available on responsible request to the corresponding author (kennec30@tcd.ie).
